# The complete chloroplast genome of *Castanopsis hystrix* Hook. f. & Thomson ex A. DC. 1863 (Fagaceae)

**DOI:** 10.1080/23802359.2023.2253999

**Published:** 2023-09-08

**Authors:** Zifan Gui, Yueqi Sun, Dong Lin, Lianxiang Zhong, Qiulan Wei, Hui Zhu, Mimi Li

**Affiliations:** a Shenzhen Park Management Center, Shenzhen, China; b Jiangsu Key Laboratory for the Research and Utilization of Plant Resources, Institute of Botany, Jiangsu Province and Chinese Academy of Sciences, Nanjing, China; c Guangxi Forestry Research Institute, Nanning, China; d Forestry College, Nanjing Forestry University, Nanjing, China

**Keywords:** *Castanopsis hystrix*, chloroplast genome, fagaceae, phylogenetic analysis

## Abstract

*Castanopsis hystrix* Hook. f. & Thomson ex A. DC. 1863 (Fagaceae) is an evergreen broad-leaved tree with high economic and ecological value. In this study, the complete chloroplast genome of *C. hystrix* was sequenced, assembled and annotated. The plastome (plastid genome) of *C. hystrix* was 160,624 bp in size, consisting of a pair of inverted repeats (IRs, 25,699 bp), a large-single-copy (LSC, 90,276 bp) region, and a small-single-copy (SSC, 18,950 bp). The overall GC content of *C. hystrix* was 36.8%. A total of 133 genes were annotated, including 88 protein-coding genes (PCG), 37 transfer RNA genes (tRNA), and eight ribosomal RNA genes (rRNA). A maximum likelihood analysis showed that the *Castanopsis* species form a monophyletic clade. *C. hystrix* is most closely related to *C. tibetana* with 100% bootstrap support value. The result enriches the genomic data for the genus *Castanopsis*, which will contribute to future studies in phylogenetics and evolution.

## Introduction

*Castanopsis hystrix* Hook. f. & Thomson ex A. DC. 1863, an evergreen broad-leaved tree belonging to the family Fagaceae, is mainly distributed in low altitude areas of tropical and subtropical Asia (Huang et al. 1999). Its timber can be used in furniture production, the shipbuilding industry, and infrastructure construction. The seeds of this species are the preferred material for making wine and feed (Zhou 2007). The abundant litterfall of *C. hystrix* helps maintain the global carbon cycle in forest ecosystems (Liu et al. 2012). Genomic characterization is the most essential step in breeding programs. However, the genomic information of *C. hystrix* is poorly understood. In this study, the chloroplast genome of *C. hystrix* was sequenced, which can provide a reference for exploration, utilization and conservation and provide basic genetic resources for further genetic and phylogenetic studies.

## Materials and methods

**Figure 1.**
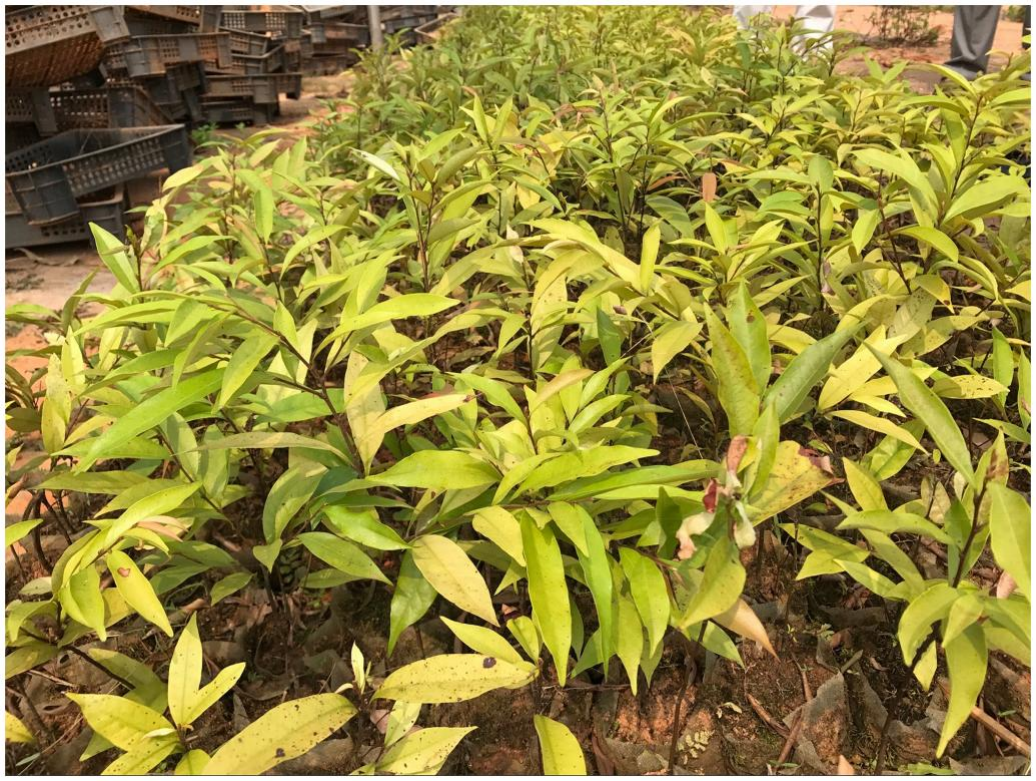
The photograph of *Castanopsis hystrix*. This image was taken by Hui Zhu at Guangxi Forestry Research Institute, Nanning Guangxi, China. *C. hystrix* is an evergreen broad-leaved tree with leaf blade margin shallowly, serrate from middle to apex, secondary veins usually not reaching margin and petiole rarely longer than 1 cm.

Fresh and mature leaves of *Castanopsis hystrix* (Figure 1) were obtained with permission from the Guangxi Forestry Research Institute (latitude 22.93 N, longitude 108.36E), and voucher specimen (collection number: 20210317001) was kept at the Guangxi Forestry Research Institute (http://www.gxlky.com.cn/index.php?siteid=6, Hui Zhu, gfrizhuhui@163.com). Total genomic DNA was extracted using a DNeasy Plant Mini Kit (Qiagen, Germany). The library with insert sizes of 300–500 bp was constructed and sequenced on the Illumina HiSeq X Ten platform (San Diego, USA) at Novogene (Beijing, China). Approximately 2 Gb of clean data was obtained after filtering the adaptors and low-quality bases sequences by Trimmomatic (Bolger et al. 2014). NOVOPlasty (Dierckxsens et al. 2017) was employed to *de novo* assemble the paired-ends reads. The sequencing depth coverage was conducted by Geneious (Kearse et al. 2012). The chloroplast genome annotations of *C. hystrix* were generated by GeSeq (https://chlorobox.mpimp-golm.mpg.de/geseq.html, Tillich et al. 2017) and adjusted manually using Geneious. The map of chloroplast genome, cis-splicing genes and trans-splicing gene of *C. hystrix* was drawn by CPGview (http://www.1kmpg.cn/cpgview/, Liu et al. 2023). Full plastomes of 19 species were aligned by MAFFT (Katoh and Standley 2013), of which 18 were retrieved from the NCBI database. Two taxa, namely, *Quercus aquifolioides* (KX911971) and *Q. tungmaiensis* (MF593893), were chosen as outgroups according to a previous study (Ye et al. 2019), the maximum likelihood (ML) tree was constructed in RAxML (Stamatakis 2014) with 1000 bootstrap replicates and the best-fitting GTR + GAMMA model to investigate the phylogenetic relationships of *C. hystrix* within the genus *Castanopsis*.

## Results

**Figure 2.**
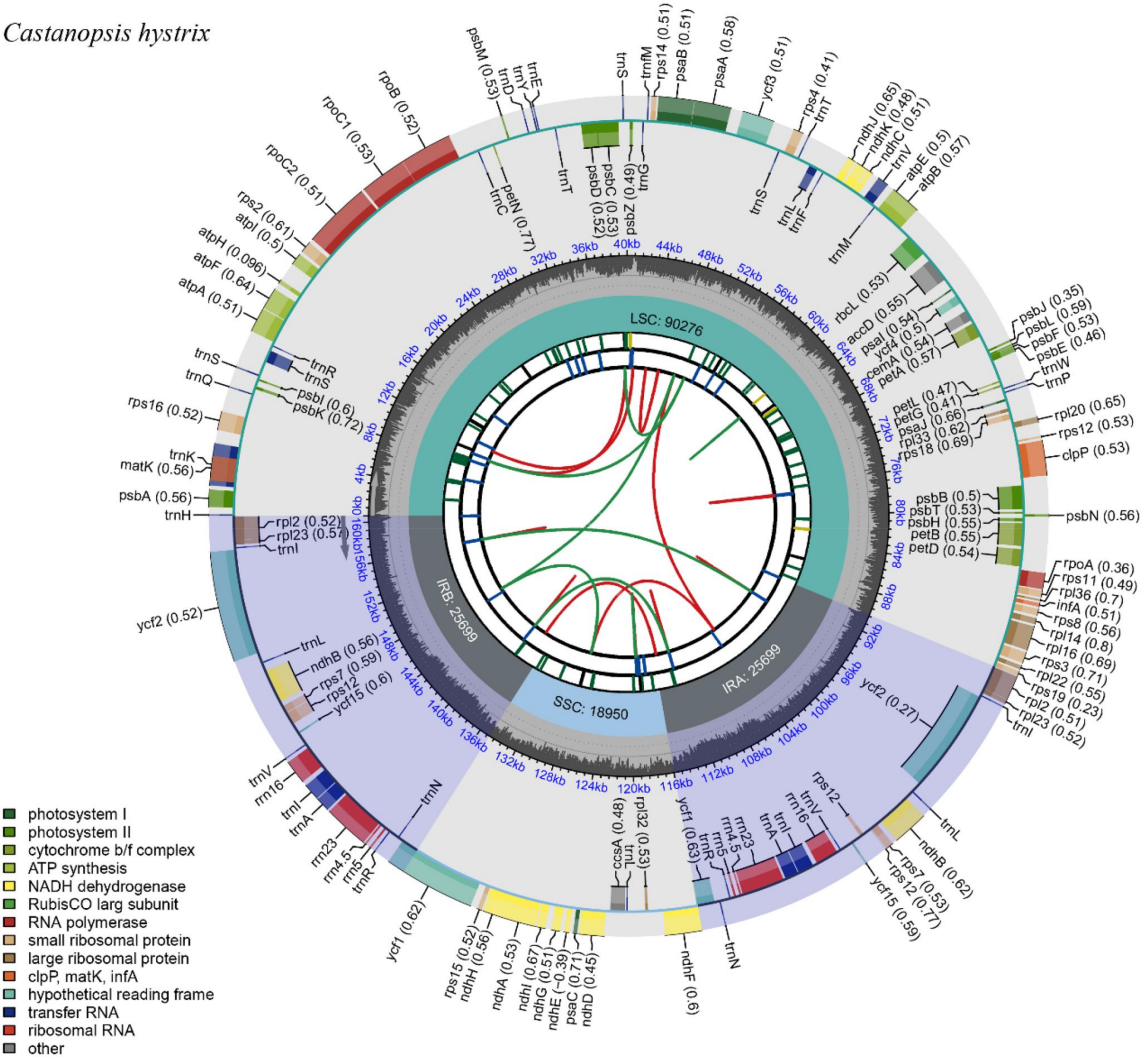
The complete chloroplast genome map of *Castanopsis hystrix*, which was generated by CPGview. LSC, SSC, and IR (IRa and IRb) with their length are represented on the first circle. The second circle shows the GC ratio in dark gray. The third circle displays the genes with the colors based on their functional classification presented at the left of the circular map. Genes located on the inner are transcribed in a clockwise, and those outer of circle are transcribed in an anticlockwise.

A total of 740,890 reads were assembled to the complete plastid genome of *Castanopsis hystrix* with an average coverage of 692 (Figure S1). The assembled plastid genome was finally deposit to the NCBI (National Center for Biotechnology Information, https://www.ncbi.nlm.nih.gov/) with accession number ON184044. The plastome possesses a quadripartite structure with 160,624 bp in length, consisting of a pair of inverted repeats (IRs, 25,699 bp), a large-single-copy (LSC, 90,276 bp) region, and a small-single-copy (SSC, 18,950 bp) (Figure 2). The plastome of *C. hystrix* was AT-rich, and the overall GC content was 36.8%, with that of IRs, SSC and LSC at 42.8%, 34.6%, and 30.9%, respectively. A total of 133 genes were annotated, including 88 protein-coding genes (PCG), 37 transfer RNA genes (tRNA), and eight ribosomal RNA genes (rRNA). Of these, 18 genes were duplicated in IR regions, including 7 PCGs (*rpl*2, *rpl*23, *ycf*2, *ndh*B, *rps*7, *rps*12 and *ycf*15), 7 tRNA genes (*trn*I-CAT, *trn*L-CAA, *trn*V-GAC, *trn*I-GAU, *trn*A-UGC, *trn*R-ACG and *trn*N-GTT), and 4 rRNA genes (*rrn*16, *rrn*23, *rrn*4.5 and *rrn*5). In addition, we detected 11 cis-splicing (intron removal) genes, namely *rps*16, *atp*F, *rpo*C1, *ycf*3, *clp*P, *pet*B, *pet*D, *rpl*16, *rpl*2(*2), *ndh*B(*2), and *ndh*A (Figure S2) and one trans-splicing (spliced leader addition) genes *rps*12 (Figure S3).

**Figure 3.**
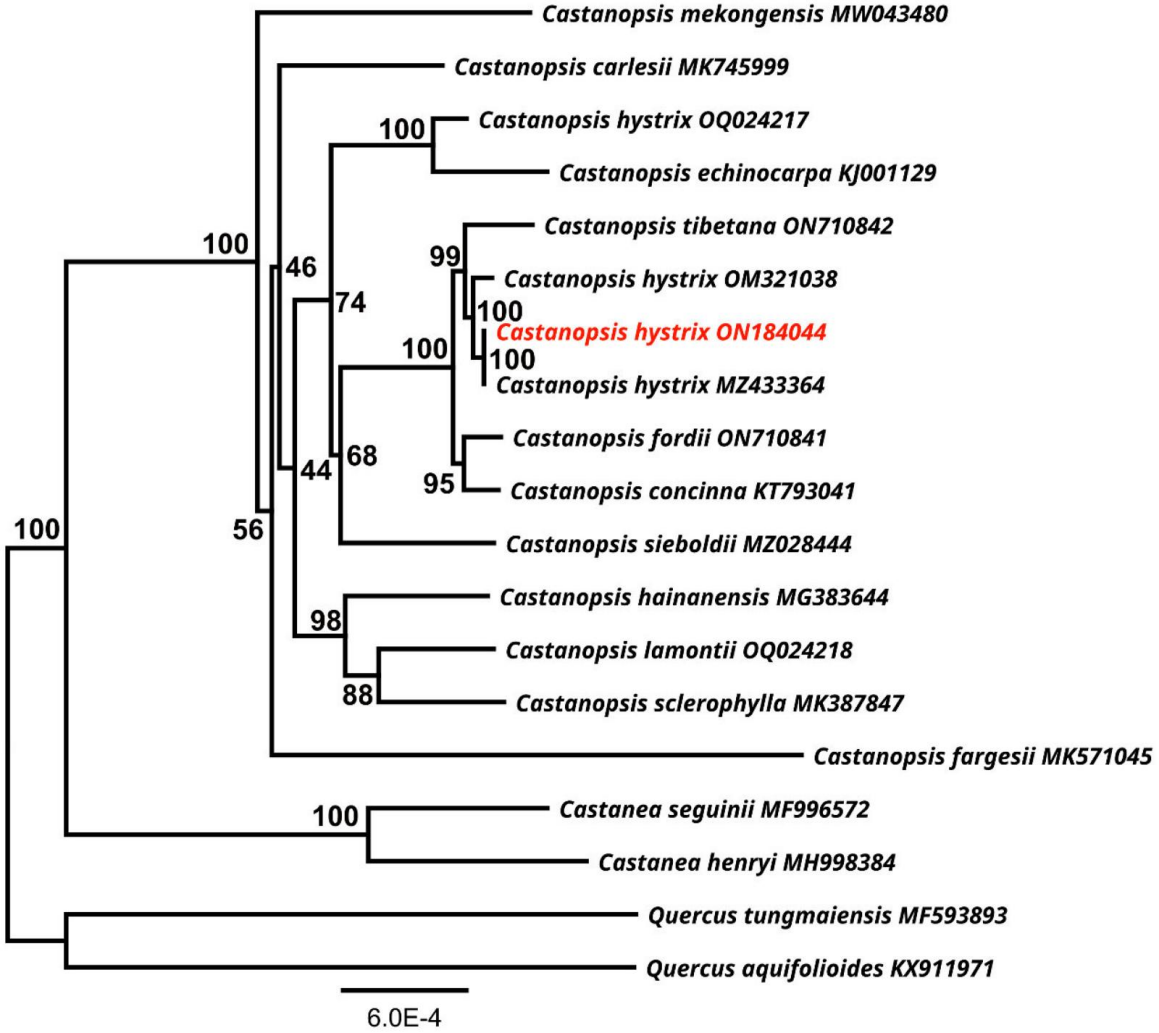
A maximum-likelihood (ML) based phylogenetic tree of *Castanopsis hystrix* using *Quercus aquifolioides* and *Q. tungmaiensis* as outgroups. The scale bar represents the number of nucleotide substitutions per site. The numbers on each node indicated the ML bootstrap support with 1000 replicates. The following sequences were used: *Castanea seguinii* MF996572, *Castanea henryi* MH998384 (Gao et al. 2019), *Castanopsis carlesii* MK745999 (Sun et al. 2019), *Castanopsis concinna* KT793041, *Castanopsis echinocarpa* KJ001129, *Castanopsis fargesii* MK571045 (Ye et al. 2019), *Castanopsis fordii* ON710841 (Wang et al. 2023), *Castanopsis hystrix* OQ024217, *Castanopsis hystrix* ON184044 (this study), *Castanopsis hystrix* OM321038, *Castanopsis hystrix* MZ433364, *Castanopsis sieboldii* MZ028444 (Park et al. 2021), *Castanopsis hainanensis* MG383644 (Chen et al. 2018), *Castanopsis mekongensis* MW043480 (Peng et al. 2021), *Castanopsis sclerophylla* MK387847 (Ye et al. 2019), *Castanopsis tibetana* ON710842, *Quercus aquifolioides* KX911971 (Yang et al. 2018), *Quercus tungmaiensis* MF593893 (Yang et al. 2018).

A maximum likelihood phylogenetic analysis shows the genus *Castanopsis* forms a monophyletic clade (Figure 3). Three different accessions of *C. hystrix* (ON184044, MZ433364, OM321038) are clustered together as a sister group to *C. tibetana* (ON710842), with a statistical value of 100%. However, one *C. hystrix* (OQ024217) sample is grouped together with *C. echinocarpa* (KJ001129). In addition, the sister species pairs, *C. hainanensis* (MG383644), *C. lamontii* (OQ024218) and *C. sclerophylla* (MK387847), *C. concinna* (KT793041) and *C. fordii* (ON710841) had 98% and 95% support, respectively.

## Discussion and conclusion

*Castanopsis* is one of the three largest genera in the Fagaceae family (Huang et al. 2023). However, the phylogenetic relationship and species identification remain poorly resolved due to phenotypic diversity, coupled with hybridization issue. Plastome data could be an ideal tool in resolving the phylogenetic relationships and species identification. *Castanopsis hystrix* is of great economic and ecological value. In this study, the chloroplast genome of *C. hystrix* has been sequenced, assembled and annotated. The structural characteristics of *C. hystrix* is similar to most angiosperm, which shows the typical four parts, consisting of a pair of inverted repeats, a large-single-copy region, and a small-single-copy (Palmer 1985). By comparing with previously known genomes of *C. hystrix*, the high-level conserved chloroplast genome was confirmed which could be demonstrated as a super-DNA barcode to distinguish *C. hystrix* from the related *Castanopsis* species. Additionally, the presumably misidentification of *C. hystrix* (OQ024217) was also recognized in the present study. Moreover, the phylogenetic position of *C. hystrix* in the genus *Castanopsis* has been clearly resolved. The result enriches the genomic data for the genus *Castanopsis*, which will contribute to phylogenetic and evolutionary studies in future.

## Supplementary Material

Supplemental Material

## Data Availability

The genome sequence data that support the findings of this study are openly available in GenBank of NCBI at (https://www.ncbi.nlm.nih.gov/) under the accession number ON184044. The associated BioProject, SRA, and Bio-Sample numbers are PRJNA828973, SRR18843872 and SAMN27681347, respectively.
