# Supplementary material for: The complete chloroplast genome of Castanopsis hystrix Hook. f. & Thomson ex A. DC. 1863 (Fagaceae)
Source: Mitochondrial DNA B Resour. 2023 Sep 8;8(9):956–9. doi: 10.1080/23802359.2023.2253999 (PMC10494720; doi:10.1080/23802359.2023.2253999)

**Supplemental material**

Figure S1. The sequencing depth of coverage plots of *Castanopsis hystrix*.


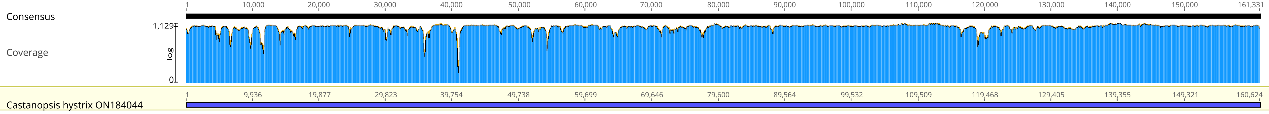


Figure S2. The map of cis-splicing genes of *Castanopsis hystrix*.


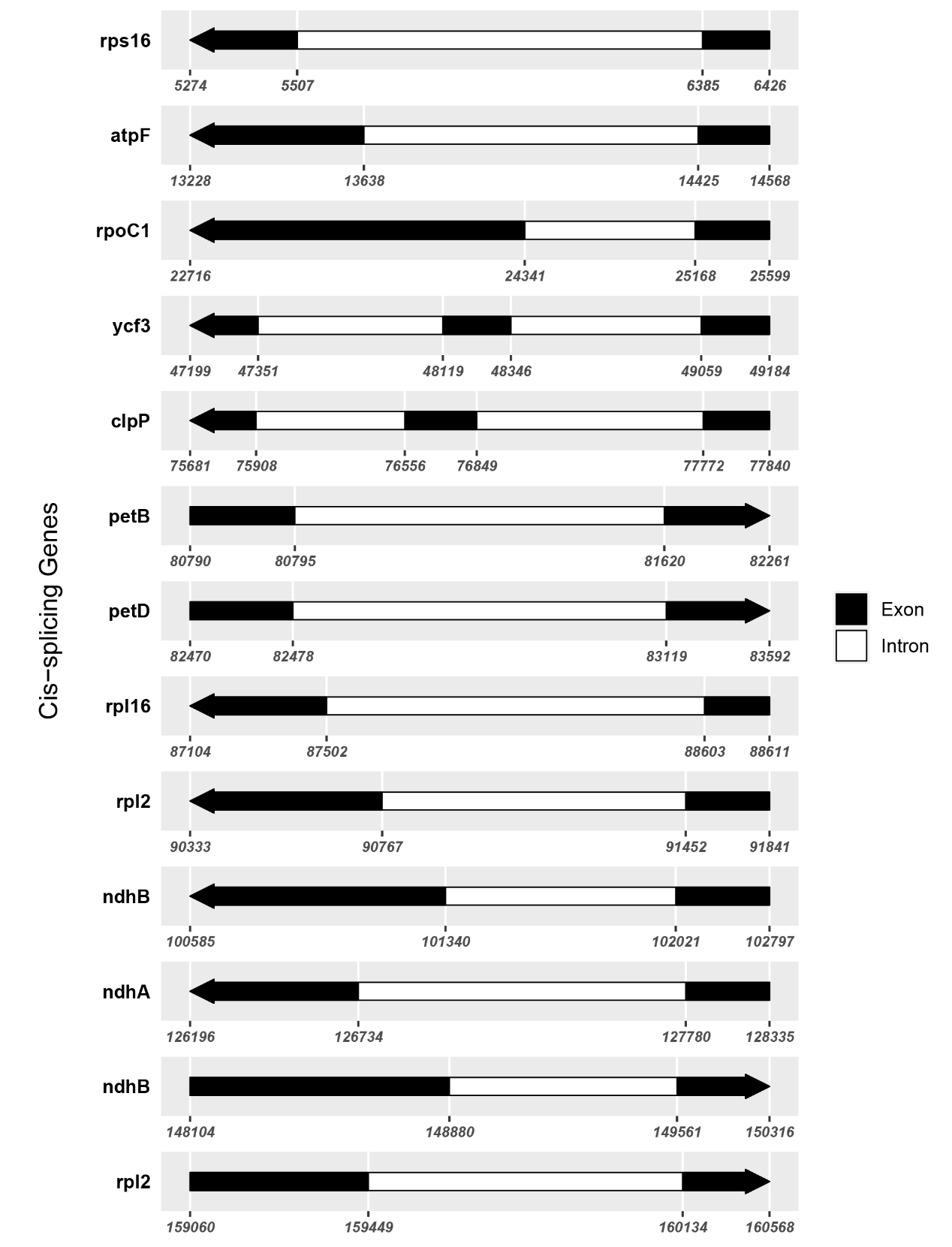


Figure S3. The map of trans-splicing gene *rps*12 of *Castanopsis hystrix*.


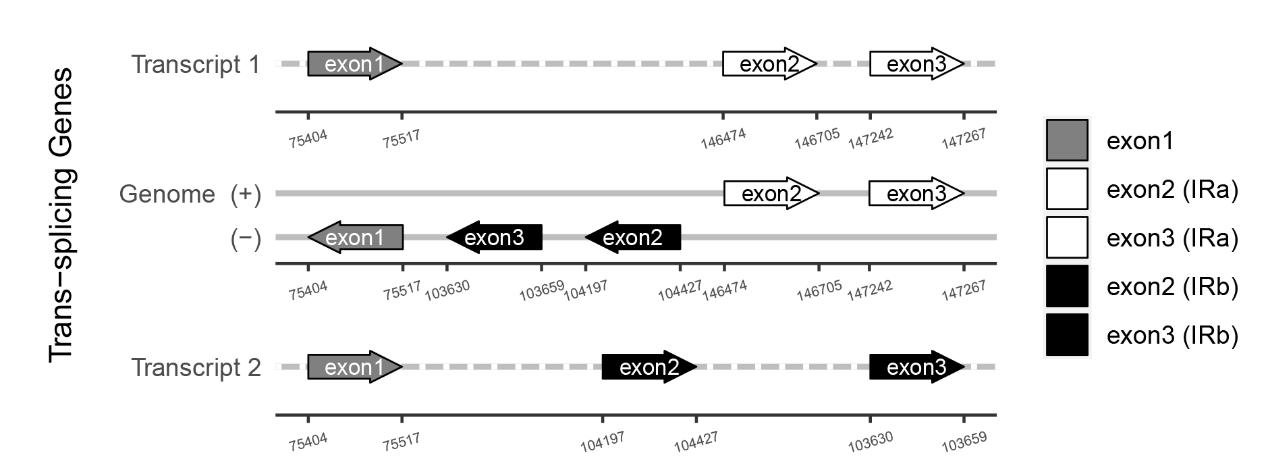

Supplement: Supplemental Material [file TMDN_A_2253999_SM5148.docx]
